# Pure Uterine Lipoma on 18FDG PET/CT: Rare But Easy to Diagnose

**DOI:** 10.7759/cureus.4334

**Published:** 2019-03-27

**Authors:** Maseeh U Zaman, Nosheen Fatima, Wasim A Memon, Areeba Zaman, Sidra Zaman

**Affiliations:** 1 Radiology, Aga Khan University Hospital, Karachi, PAK; 2 Internal Medicine, Dr. Ruth K.M. Pfau Hospital, Karachi, PAK; 3 Radiology, Dow Medical College, Karachi, PAK

**Keywords:** pure lipoma of uterus, lipomatous tumor of the uterus, 18-fdg pet/ct

## Abstract

Tumors of the uterus are extremely uncommon. Pure lipomas of the uterus are very rare, and only a few cases have been reported. We are presenting a case of a post-menopausal lady, a survivor of right breast cancer who had an 18 F-fluorodeoxyglucose positron emission tomography/computed tomography (^18^FDG PET/CT) for evaluation of a suspected right lung nodule. The scan was negative for hypermetabolic abnormality. However, a hypodense and non-metabolic lesion was seen in the fundus of the uterus. On subsequent hysterectomy, it was found to be a pure lipoma.

## Introduction

Lipomatous tumors of the uterus are very uncommon (incidence: 0.03% - 0.25%) [1] and were first described by Lobstein in 1816 [2]. Most reported cases have been of the mixed type containing varying amounts of smooth muscle and fibrous elements. Pure lipomas involving the uterine wall are exceedingly rare; the exact incidence is not known as only a few cases have been reported thus far in the literature [3]. Because of the pure fatty nature of the tumor, computerized tomography (CT) is considered the diagnostic modality [4]. In current oncological practice, F-18 fluorodeoxyglucose (^18^FDG)-based positron emission tomography and CT (PET/CT) has become an integral part of management. Lipomas consistently show a low ^18^FDG uptake (standardized uptake value (SUV) < 2.0) [5], but current literature is silent about the features of a pure lipoma of the uterus using ^18^FDG PET/CT imaging. We are presenting the first case of a pure uterine lipoma found on ^18^FDG PET/CT imaging of a postmenopausal breast cancer survivor.

## Case presentation

**Figure 1:**
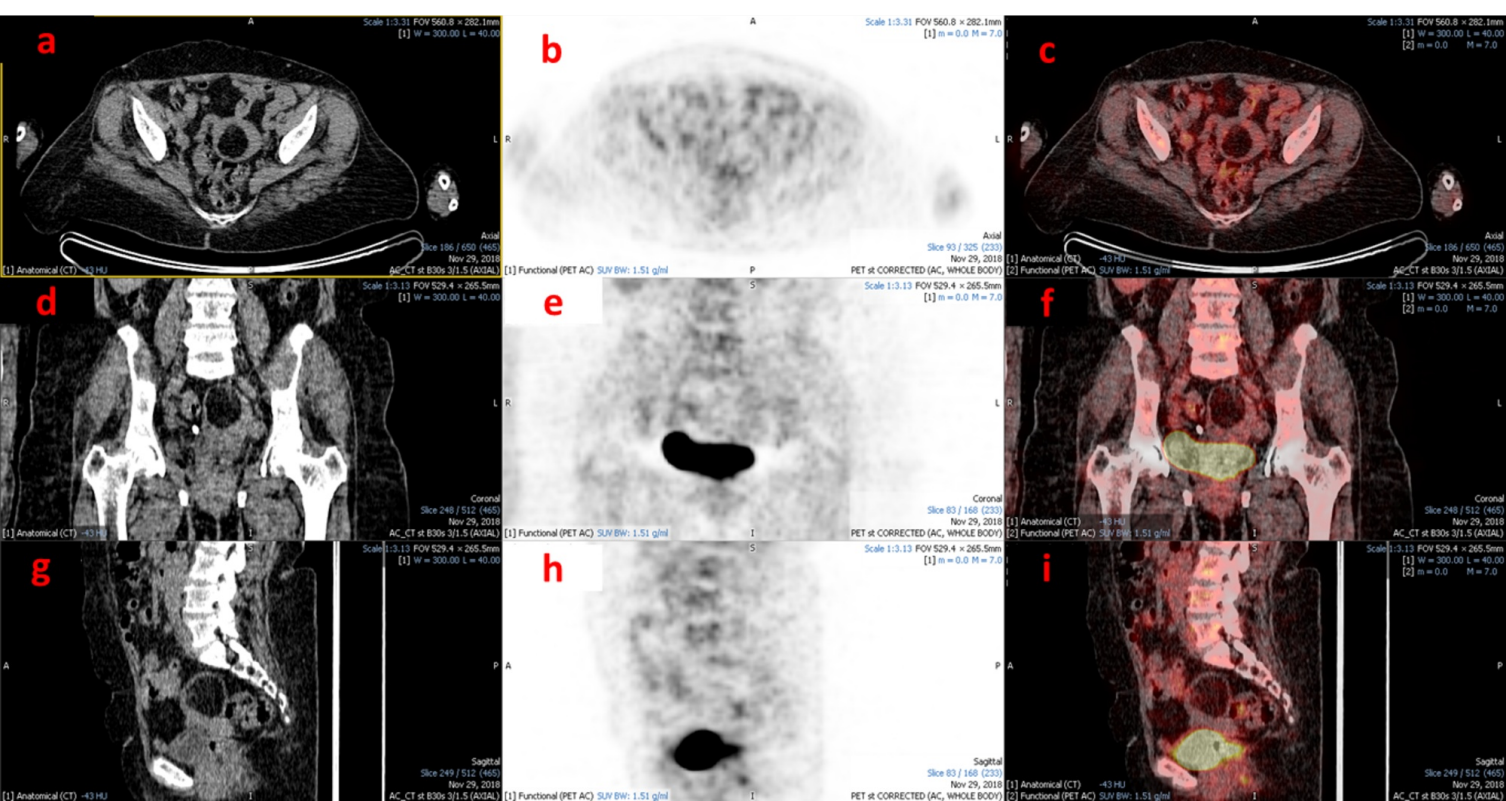
18FDG PET/CT images (a-c: axial; d-f: coronal; g-i: sagittal) showing a well-defined hypodense area (Hounsfield unit (HU): -89.9) without metabolic activity in the uterine fundus .

Herein, we present the case of a 67-year-old female breast cancer survivor who had a right lumpectomy for ductal carcinoma in 2012. She was clinically asymptomatic. However, a recent CT scan of the chest revealed a subcentimeter soft tissue nodule in the lower lobe of the right lung which was not appreciable on previous CT lung window films (no soft images were available). It was considered a possible granuloma but an ^18^FDG PET/CT was recommended for evaluation and surveillance of the nodule. The PET/CT was performed with 10.2 mCi of ^18^FDG after 60-minute skull to mid-thigh images were acquired using a low-dose, non-contrast enhanced CT protocol. The scan showed no morphological or functional evidence of tumor in either breast or hypermetabolic nodal and no hepatic, adrenal, pulmonary, or bony metastases. There was a redemonstration of the subcentimeter ametabolic soft tissue nodule in the lower lobe of the right lung with minimal infiltrate in the right lung base. The uterine fundus revealed an ^18^FDG non-avid hypodense area (Hounsfield unit (HU): -89.9) measuring 40 x 30 mm (anteroposterior (AP) and transverse (TV) dimensions) without regional nodes (Figure 1). Based on the image characteristics, it was reported as a lipoma. An uneventful hysterectomy was performed after three weeks on request by the family, and the histopathology revealed a pure lipoma.

## Discussion

A pure lipoma of the uterus is an exceedingly rare tumor and few cases have been published thus far. The first reported case of a pure uterine lipoma on preoperative ultrasound was published in 1979 [6] and the authors correctly identified the fatty nature of the tumor but incorrectly reported it as dermoid. The first case of a pure lipoma of the uterus identified on CT scan was reported in 1988 which had well-circumscribed and homogenously hypodense attenuation parameters (HU: -118) [7]. However, mixed uterine lipomas may mimic fibroid tumors depending upon the contribution of non-lipomatous tissue. Because of these peculiar features, a CT scan is considered as the imaging modality of choice for uterine pure lipomas. Lipomas (white fat tumors) are considered to have consistently low ^18^FDG uptake in contrast to hibernomas (brown fat tumors), which are usually hypermetabolic, as they are thermogenic and rich in mitochondria [5]. Uterine tumors show a wide spectrum of ^18^FDG uptake ranging from low uptake in benign tumors (some leiomyomas may have high uptake) to intense uptake in malignant neoplasms [8]. However, the literature is silent about ^18^FDG PET/CT imaging of pure lipoma of the uterus. To the best of our research, this is the first case report revealing the ^18^FDG PET/CT imaging features of a pure lipoma of the uterus.

Pure lipomas of the uterus are usually found in post-menopausal women and about 60% are intramural. They are usually asymptomatic but may present with symptoms like pain and bleeding, particularly if associated with uterine leiomyomas. On ultrasonography, these tumors have an echogenic center due to the fat and are avascular with a hypoechoic rim due to myometrium in the periphery [9]. On magnetic resonance imaging (MRI), a uterine lipoma would appear as a high signal on T1 and T2 sequences and show signal dropout on fat, thus confirming the diagnosis [9]. On CT examination, these are classically hypodense with a hyperdense periphery when they are intramural [7]. In this case report, the CT appearance of a pure lipoma of the uterus was also similar to the previous report with an ametabolic (^18^FDG non-avid) appearance on PET images (hypodense and hypometabolic) as commonly observed in non-uterine pure lipomas. With the increasing use of ^18^FDG PET/CT in oncology, there are high odds of finding more cases of pure uterine lipomas and these characteristic imaging features would help the reporting physicians to make a preoperative diagnosis with higher confidence.

## Conclusions

A pure lipoma of the uterus is an exceedingly rare benign tumor, and this first case report depicting its appearance on ^18^FDG PET/CT imaging would certainly help the reporting nuclear physicians and radiologists to diagnose it with a high level of confidence.
